# Environmental co-exposure to organophosphate and pyrethroid pesticides and mental health status in rural communities near an industrial pig farming facility

**DOI:** 10.1038/s41598-026-40098-1

**Published:** 2026-02-18

**Authors:** Rocío Hojas, José Norambuena, América Ponce, Joaquín Toro, Jandy Adonis-Rojas, Sebastián Pozo, Bárbara Figueroa, Francisca Cabezas, Cristian Valdés, Liliana Zúñiga-Venegas, Benjamín Castillo, Natalia Landeros, Boris Lucero, Ramón D. Castillo, Cynthia Carrasco, Juan Pablo Gutiérrez-Jara, Catalina Saavedra, Patricio Yáñez, María Ignacia Valdés, María Victoria Rodríguez, Andrés Canales-Johnson, Tristan Bekinschtein, María Teresa Muñoz-Quezada

**Affiliations:** 1 School of Medicine, Faculty of Medicine, Universidad de Chile, Independencia 1027, Santiago, Chile; 2 Master in Public Health, School of Public Health, Faculty of Medicine, Universidad de Chile, Independencia 939, Santiago, Chile; 3 Medical Specialty in Public Health, Faculty of Medicine, Universidad de Chile, Independencia 1027, Santiago, Chile; 4 School of Biotechnology Engineering, Faculty of Agricultural and Forestry Sciences, Universidad Católica del Maule, Avenida San Miguel 3605, Talca, Chile; 5 Centro de Investigación de Estudios Avanzados del Maule (CIEAM) , Universidad Católica del Maule, Avenida San Miguel 3605, Talca, Chile; 6 Department of Agricultural Production, Faculty of Agricultural Sciences, Universidad de Chile, Avenida Santa Rosa 11315, La Pintana, Santiago, Chile; 7 Facultad de Medicina, Universidad Católica del Maule, Avenida San Miguel 3605, Talca, Chile; 8 Research Center in Neuropsychology and Cognitive Neurosciences (CINPSI Neurocog), Faculty of Health Sciences, Universidad Católica del Maule, Avenida San Miguel 3605, Talca, Chile; 9 Centro de Investigación en Ciencias Cognitivas, Facultad de Psicología, Universidad de Talca, Avenida Lircay s/n, Talca, Chile; 10 Doctorate in Psychology, Faculty of Health Sciences, Universidad Católica del Maule, Avenida San Miguel 3605, Talca, Chile; 11 Consciousness and Cognition Lab, Department of Psychology, University of Cambridge, Downing Street, Cambridge, CB2 3EB UK; 12 School of Public Health, Faculty of Medicine, Universidad de Chile, Independencia 939, Santiago, Chile; 13 Centro para la Prevención y el Control del Cáncer (CECAN), Santiago, Chile; 14 Centro de Investigación y Acción en Determinación Social y Salud Mental (CIADES), Santiago, Chile; 15 Advanced Center for Chronic Diseases (ACCDiS), Santiago, Chile; 16 School of Obstetrics and Childcare, Faculty of Health Science, Universidad Católica del Maule, Talca, Chile; 17 Universidad de Talca, Talca, Chile

**Keywords:** Pesticides, Depression, Anxiety, Environmental exposure, Mental health, Environmental sciences, Risk factors

## Abstract

Chronic exposure to pesticide residues from large-scale agro-livestock operations remains insufficiently characterized, particularly among rural populations living near industrial pig farming facilities. This study examined the association between co-exposure to organophosphate and pyrethroid pesticide residues in soil and well water and mental health outcomes among adults residing near an industrial pig farming facility in rural Chile. A cross-sectional study was conducted with 82 adults. Peridomestic soil and well water samples were analyzed using gas chromatography–tandem mass spectrometry to detect five pesticides: chlorpyrifos, diazinon, pirimiphos-methyl, cypermethrin, and lambda-cyhalothrin. Mental health outcomes included depressive symptoms, anxiety, emotional affect, and health-related quality of life, assessed with validated instruments. Robust linear regression models were used, adjusting for age, sex, education level, and body mass index. Higher chlorpyrifos concentrations in water were associated with increased depressive symptom scores (β = 0.180; 95% CI: 0.016, 0.345) and lower mental health-related quality of life (β = −0.713; 95% CI: −1.288, − 0.137). Cypermethrin concentrations in water were associated with higher psychological distress (β = 0.324; 95% CI: 0.124, 0.525). In soil, pirimiphos-methyl was positively associated with depressive symptoms (β = 21.29; 95% CI: 1.78, 40.79), whereas cypermethrin showed an inverse association (β = −3.66; 95% CI: −6.99, − 0.33). Lambda-cyhalothrin concentrations in soil were inversely associated with overall quality of life (β = −15.13; 95% CI: −27.42, − 2.84). Male sex was positively associated with overall quality of life (β = 14.96; 95% CI: 3.14, 26.79). These findings indicate associations between environmental pesticide residues in soil and water and multiple dimensions of mental health among rural populations living near industrial pig farming operations. Longitudinal studies are needed to clarify temporal relationships and potential cumulative effects.

**Supplementary Information:**

The online version contains supplementary material available at 10.1038/s41598-026-40098-1.

## Introduction

The worldwide expansion of pesticide use has increased agricultural productivity, while pesticide exposure has also been associated with effects on human health and the environment. Exposure to pesticides, particularly organophosphates, has been linked to acute and chronic neurotoxic outcomes, as well as to psychiatric symptoms including depression, anxiety, and suicidal ideation^1–5^. Pesticide self-poisoning accounts for an estimated 14 to 20% of global suicides, corresponding to more than 110,000 deaths annually, with a higher burden in low- and middle-income countries where highly hazardous pesticides remain widely accessible^4,6^.

### Environmental and non-occupational pesticide exposure

Environmental pesticide exposure is common in rural settings. Non-occupational exposure occurs through pathways such as drift from nearby agricultural fields, contamination of household water sources, and the residues in peridomestic soil and dust^6,7^. Studies show that individuals not directly involved in pesticide application, including women, children, and older adults, often have detectable urinary pesticide metabolites, commonly associated with residential proximity to treated crops or domestic insecticide use^8,9^.

These findings indicate the relevance of residential and environmental sources of exposure, particularly in areas with intensive agro-industrial activity. Monitoring pesticide residues in soil and water may provide a practical proxy for cumulative environmental exposure in settings with limited biomonitoring capacity^6^.

### Evidence from Latin America and the Caribbean

In Latin America and the Caribbean, systematic reviews have reported associations between pesticide exposure and a range of adverse health outcomes, including genotoxic damage, neurobehavioral impairments, reproductive and endocrine disorders, pregnancy complications, and increased cancer risk^10^. The populations most frequently examined in this literature are agricultural workers and children, groups that are predominantly exposed to organophosphates, carbamates, herbicides, and other pesticide compounds^11^. Chronic exposure may occur through pathways such as aerial dispersion, domestic application, occupational contact, and ingestion of contaminated food or water^12^. Environmental health surveillance systems in the region are limited in scope and technical capacity, with constraints on detection and response, especially in rural areas^13^.

A recent meta-analysis reported a statistically significant association between pesticide poisoning and depressive symptoms (OR = 2.94; 95% CI = 1.79–4.83; *p* < 0.001)^14^, whereas non-acute exposure was not associated with increased risk. At the biochemical level, organophosphates inhibit acetylcholinesterase (AChE) and are also associated with mechanisms such as oxidative stress, neuroinflammatory processes, mitochondrial dysfunction, and alterations in serotonergic and glutamatergic neurotransmission, which have been implicated in the neurobiology of depression^1,15–18^. These mechanisms may affect brain regions, including the amygdala and hippocampus, which are involved in emotion regulation and the stress-related response^17^.

Long-term occupational exposure to organophosphates has been associated in several studies with a higher frequency of psychiatric symptoms, including anxiety, suicidal ideation, and cognitive impairment, even in the absence of acute intoxication episodes¹^[,18^. In addition, national regulatory interventions, such as restrictions or bans on specific highly hazardous pesticides in countries including Sri Lanka and South Korea, have been associated with reductions in suicide rates (37% to 70%), while agricultural production indicators were maintained during the same periods^3,5^.

### Evidence from Chile

In Chile, the Maule Region, located in the south-central zone, is characterized by high levels of agricultural activity and pesticide use^19^. It ranks second nationally in agrochemical sales (15.7%) and in cultivated land area, following the O’Higgins and Araucanía regions, respectively^20^. National surveillance data indicate that in 2023, the Maule Region accounted for 25% of all reported cases of acute pesticide poisoning^21^. Studies conducted in the region have documented extensive environmental exposure affecting both children and adults. A recent longitudinal study reported an association between urinary levels of diethyl alkyl phosphate (DEAP) metabolites, biomarkers of organophosphate exposure, and depressive symptoms among male agricultural workers, with a dose–response pattern observed even at exposure levels below clinical thresholds^22^.

In the Coquimbo Region, a semi-arid area in north-central Chile with intensive fruit and vegetable production, studies describe chronic health effects associated with pesticide exposure, including genotoxic alterations, neurocognitive impairments, and increased oxidative stress markers in agricultural workers and residents living near treated fields^13,23,24^. Similar patterns have been described in other regions of central Chile, involving both occupational and environmental exposure contexts.

The rural area known as “El Arbolillo,” which includes sectors of San Javier and Cauquenes, is characterized by the presence of an industrial pig farming operation and surrounding agricultural activities. Since the beginning of operations, residents have reported persistent odors, increased fly density, and intensified insecticide use in nearby households.

During the preliminary phase of this study, more than 200 administrative, legal, and technical documents were reviewed. These records indicated that organophosphates (diazinon, pirimiphos-methyl) and pyrethroid (cypermethrin) pesticides were systematically applied as part of vector control practices^25^. These practices have been associated with repeated environmental exposure and have prompted official inspections and collective actions by local residents.

### Study objective

The aim of this study was to examine the association between co-exposure to organophosphate and pyrethroid pesticide residues in peridomestic soil and well water and mental health outcomes among adults residing near an industrial pig farming facility in rural Chile.

This study provides data on the relationship between chronic simultaneous exposure to organophosphate and pyrethroid residues in environmental matrices and mental health outcomes among rural populations not occupationally involved in pesticide application. It addresses limited evidence on environmental co-exposure to these pesticide classes and their potential association with mental health outcomes, particularly in rural populations living near industrial livestock operations. This evidence is relevant in Chile and in other Latin American or low- and middle-income countries, where regulatory frameworks and environmental monitoring systems remain constrained.

## Results

A total of 95 individuals were recruited, and 82 completed the mental health assessments and were included in the analysis. The sample included 44 men (54.1%) and 38 women (45.9%), with an average age of 52.4 years (SD 15.2) and a range of 18 to 78 years. Marital status showed that 54.1% were married, 25.9% were single, 9.4% were cohabiting, 8.2% were separated or divorced, and 2.4% were widowed. For analysis, participants were classified as either living with a partner (married or cohabiting; 63.5%) or not living with a partner (36.5%).

In terms of educational attainment, 31.8% had completed primary education or less, 35.3% had some secondary education, and 32.9% had higher education (technical or university). For analytical purposes, education was grouped into two categories: 52.9% had not completed secondary education, and 47.1% had completed secondary or higher education.

The mean monthly per capita income was CLP $205,302 (SD = $133,998), equivalent to approximately USD $217 (SD = $141) at an exchange rate of 945 CLP/USD (April 2025). Reported incomes ranged from CLP $25,000 (USD $26) to CLP $666,666 (USD $705). The average length of residence in the area was 16.3 years (SD = 21.5).

With respect to tobacco use, 67.1% of participants were classified as the low-use category, 28.2% within the moderate-use group, and 4.7% within the high-use category. For alcohol consumption, 89.4% were in the low-use group, 8.2% in the moderate-use group, and 2.4% in the high-use category. Overall, problematic substance use levels in adults were low.

In the combined analysis of peridomestic soil and household well water samples (Table 1), multiple pesticide residues were identified. In soil (µg/kg), detection frequencies were 57.32% for chlorpyrifos, 13.41% for diazinon, 9.76% for lambda-cyhalothrin, 7.32% for pirimiphos-methyl, and 4.28% for cypermethrin. In well water samples (µg/L), intended for human consumption or irrigation, detection rates were 39.47% for chlorpyrifos, 25% for cypermethrin, 22.37% for diazinon, 15.79% for pirimiphos-methyl, and 9.21% for lambda-cyhalothrin.

**Table 1:** Measured concentrations of pesticide residues in peridomestic soil (µg/kg) and well water samples for human consumption and irrigation (µg/L)*.

							Percentiles			
Pesticide residues in peridomestic soil samples	Mean	Median	SD	Min	Max	% of samples > LOD	25th	50th	75th	95th
Cypermethrin	0.46	0.25	1.17	0.25	7.76	4.28	0.25	0.25	0.25	0.25
Chlorpyrifos	5.37	3.30	12.96	0.04	84.02	57.32	0.04	3.30	6.02	13.23
Diazinon	0.43	0.01	1.15	0.01	4.27	13.41	0.01	0.01	0.01	3.98
Pirimiphos-methyl	0.17	0.06	0.49	0.06	2.85	7.32	0.06	0.06	0.06	0.35
Lambda-cyhalothrin	0.55	0.32	0.74	0.32	3.71	9.76	0.32	0.32	0.32	2.67

							Percentiles			
Pesticide residues in well water samples	Mean	Median	SD	Min	Max	% of samples > LOD	25th	50th	75th	95th
Cypermethrin	1.80	0.25	2.83	0.25	8.62	25.00	0.25	0.25	0.25	8.32
Chlorpyrifos	8.08	0.04	15.20	0.04	61.57	39.47	0.04	0.04	9.09	54.19
Diazinon	0.42	0.01	0.95	0.01	4.58	22.37	0.01	0.01	0.01	2.17
Pirimiphos-methyl	0.22	0.06	0.36	0.06	1.25	15.79	0.06	0.06	0.06	1.06
Lambda-cyhalothrin	0.70	0.32	1.13	0.32	4.13	9.21	0.32	0.32	0.32	4.05

* Soil samples were collected around the perimeter of participant households, and water samples correspond to sources used for drinking and/or irrigation at participant households.

Chlorpyrifos was the most frequently detected compound in both soil and water. The mean soil concentration was 5.37 µg/kg, with a range of 0.04 to 84.02 µg/kg. In water, the mean concentration was 8.08 µg/L, with a range of 0.04 to 61.57 µg/L. Cypermethrin, diazinon, pirimiphos-methyl, and lambda-cyhalothrin were detected infrequently. Median and interquartile values for these compounds were at or below the analytical limit of detection.

Residential distance to several land uses showed negative correlations with soil and water pesticide concentrations, indicating higher residue levels at shorter distances. Chlorpyrifos in water exhibited the strongest and most consistent patterns, with higher concentrations near forestry areas (Sites 2, 6, 7, and 8), vineyards (Sites 1 and 4), and cherry orchards. Spearman coefficients ranged from − 0.255 to − 0.431 (*p* < 0.01), values that fall within the range typically interpreted as weak (|ρ| < 0.30) to moderate (0.30 ≤ |ρ| < 0.50) monotonic associations. This classification indicates that although statistically significant, most associations represent low-to-moderate gradients of concentration changes with distance, consistent with processes such as drift or surface runoff (Table 2).

**Table 2:** Environmental sources associated with higher pesticide residue levels based on residential proximity*.

Exposure source	Associated pesticide	Spearman’s Rho	*p*-value
Forestry Site 2	Chlorpyrifos (water)	−0.255	0.012
Forestry Site 3	Cypermethrin (soil)	−0.228	0.018
Forestry Site 5	Diazinon (soil)	−0.187	0.043
	Lambda-cyhalothrin (soil)	−0.190	0.041
Forestry Site 6	Chlorpyrifos (water)	−0.324	0.002
	Pirimiphos-methyl (water)	−0.304	0.003
	Lambda-cyhalothrin (water)	−0.269	0.009
Forestry Site 7	Chlorpyrifos (water)	−0.407	< 0.001
	Pirimiphos-methyl (water)	−0.352	< 0.001
	Lambda-cyhalothrin (water)	−0.332	0.001
Forestry Site 8	Cypermethrin (soil)	−0.222	0.020
	Pirimiphos-methyl (soil)	−0.205	0.030
	Chlorpyrifos (water)	−0.312	0.003
Forestry Site 9	Cypermethrin (water)	−0.253	0.013
	Diazinon (water)	−0.190	0.048
Forestry Site Ce1	Diazinon (water)	−0.194	0.037
	Diazinon (soil)	−0.193	0.045
Forestry Site Ce2	Cypermethrin (water)	−0.268	0.009
	Diazinon (water)	−0.201	0.039
Vineyard 1	Diazinon (soil)	−0.184	0.046
	Diazinon (water)	−0.431	< 0.001
	Pirimiphos-methyl (water)	−0.363	< 0.001
	Pirimiphos-methyl (soil)	−0.314	0.003
Vineyard 2	Cypermethrin (soil)	−0.209	0.028
Vineryard 4	Pirimiphos-methyl (soil)	−0.202	0.032
	Chlorpyrifos (water)	−0.383	< 0.001
	Pirimiphos-methyl (water)	−0.195	0.044
	Lambda-cyhalothrin (water)	−0.194	0.045
Cherry Orchards	Chlorpyrifos (water)	−0.299	0.004
	Diazinon (water)	−0.271	0.008
	Pirimiphos-methyl (water)	−0.362	< 0.001
	Pirimiphos-methyl (water)	−0.261	0.010

* Spearman’s rank correlation coefficients were interpreted using commonly applied thresholds: values below |0.30| were classified as weak, those between |0.30| and |0.50| as moderate, and those above |0.50| as strong monotonic associations. These categories were used to contextualize the magnitude of the correlations reported in Table 2.

Cypermethrin in soil was associated with proximity to forestry Sites 3 and 8, and vineyard Site 2. In water, cypermethrin concentrations were correlated with distance from Sites 9 and Forestry Ce2. Diazinon was associated with both soil (Sites 5, forestry Ce1, and vineyard Sites 1 and 4) and water contamination (Sites 9, Forestry Ce1, Forestry Ce2, vineyards, and cherry orchards). Pirimiphos-methyl showed positive associations in both matrices near vineyard and forestry sites, and in water samples near cherry orchards. Lambda-cyhalothrin in water was associated with forestry Sites 6 and 7, vineyard Site 1, and cherry orchards, while its detection in soil was limited to Site 5 (Table 2).

Regarding non-occupational exposure, 69.0% of participants reported using insecticides in their homes. According to participants, this practice is largely driven by the need to control high fly densities associated with odors, animal waste, and slurry from the nearby industrial pig facility. Among respondents, 90.5% stated that the survey item on pesticide use in household gardens was not applicable. Among the remaining individuals, 4.8% reported using sulfur, 2.4% biopesticides, and 2.4% pyrethroids, indicating limited but varied use in domestic cultivation.

Among those reporting indoor insecticide use, pyrethroids were the most frequently cited (48.8%), followed by combinations of organophosphates and pyrethroids (10.7%), and organophosphates alone (6.0%). A small proportion (2.4%) reported other insecticides, and 32.1% indicated that the question did not apply.

Descriptive findings (Table 3) indicate moderate to high levels of positive affect among participants (mean = 36.48, SD = 6.92 on a 10–50 scale) and comparatively low levels of negative affect (mean = 17.18, SD = 7.29), according to the PANAS scale.

Depressive symptoms assessed with the CES-D yielded a mean score of 14.64 (SD = 9.26), indicating variation in symptom severity across the sample, with some participants potentially exceeding the established clinical threshold.

Health-related quality of life, measured using the SF-12, showed moderate average scores for physical (mean = 46.53) and mental functioning (mean = 45.84), with wide standard deviations (both exceeding 28 points). The composite SF-12 score averaged 49.37, close to the midpoint of the scale, again with broad dispersion.

**Table 3:** Descriptive statistics for mental health and quality of life indicators among study participants.

						Percentiles			
	Mean	Median	SD	Min	Max	25th	50th	75th	95th
Positive affect (PANAS+)	36.48	37.0	6.92	20	50	32.0	37.0	41.0	47.0
Negative affect (PANAS-)	17.18	15.0	7.29	10	45	12.0	15.0	20.0	30.0
Depressive symptoms (CES-D)	14.64	13	9.26	1	42	7.0	13.0	19.0	34.4
Physical functioning (SF-12 Physical)	46.53	49.4	28.34	0.0	100	19.3	49.4	66.3	94.0
Mental functioning (SF-12 Mental)	45.84	45.8	28.29	0.0	100	15.7	45.8	72.3	89.2
Overall functioning (SF-12 total)	49.37	50.0	29.26	0.0	100	24.1	50.0	72.3	95.02
Depression & Anxiety (GHQ-12)	2.98	2	2.74	0	11	1.0	2.0	4.0	8.8

Psychological distress, as measured by the GHQ-12, had a mean score of 2.98 (SD = 2.74), with a range of 0 to 11. This indicates that while a substantial portion of participants reported low distress, others presented elevated levels of psychological.

**Fig. 1:**
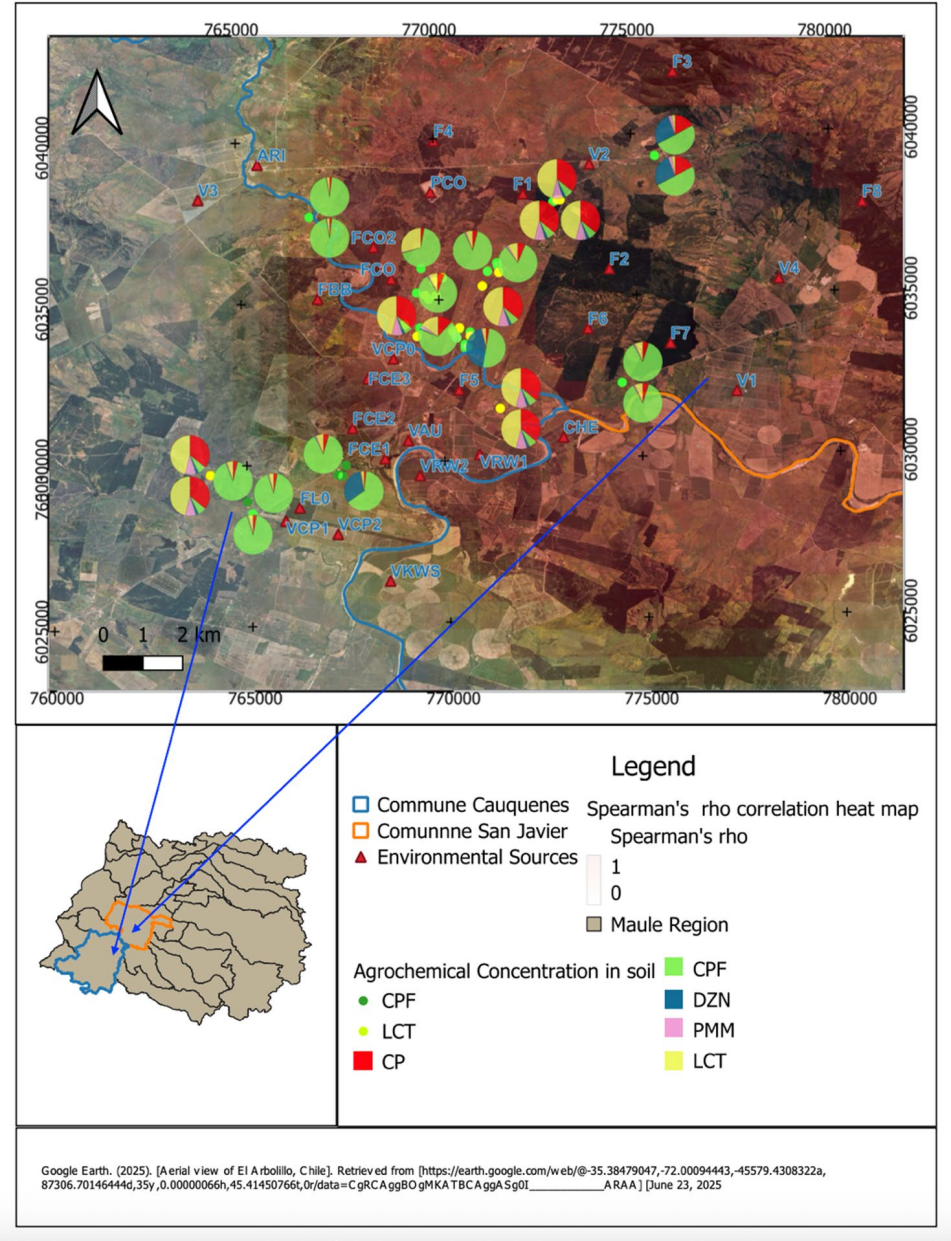
Spatial distribution of agrochemical residues in soil samples from rural households in Cauquenes and San Javier communes, Maule Region*. Map source: Google Earth satellite imagery (© Google; aerial imagery of El Arbolillo, Maule Region, Chile; accessed June 23, 2025). Map creation and editing: The authors created the maps using QGIS (version 3.34.4 ‘Prizren’; https://qgis.org). All spatial layers, symbols, and analyses were generated by the authors. *Note: Each pie chart represents the relative concentration of the five pesticides detected in peridomestic soil for a given household: chlorpyrifos (CPF), diazinon (DZN), pirimiphos-methyl (PMM), cypermethrin (CP), and lambda-cyhalothrin (LCT). The size of the pies does not indicate absolute concentration but illustrates the proportional contribution of each compound within the sample. Smaller, isolated points correspond to households with very low measured concentrations; these markers were assigned a distinct symbol solely to visually differentiate them from households with higher residue levels and do not represent a different exposure category. The small locator map contextualizes the study area within the Maule Region and identifies the positions of the Cauquenes and San Javier communes. The heat map shows the magnitude of Spearman’s rho correlations between the distance from each household to nearby agricultural and industrial sources and the levels of pesticides detected in soil. Warmer colors indicate stronger positive correlations, meaning that shorter distances to these sources were associated with higher pesticide residues, while cooler colors indicate weaker or null associations.

Figure 1 presents the spatial distribution of agrochemical residues in peridomestic soil samples from rural households in the communes of Cauquenes and San Javier, Maule Region. Each pie chart on the satellite map corresponds to a sampling site and shows the relative proportions of detected pesticides: chlorpyrifos (CPF), diazinon (DZN), pirimiphos-methyl (PMM), cypermethrin (CP), and lambda-cyhalothrin (LCT). 

The distribution is spatially heterogeneous, with higher concentrations and more varied mixtures in areas located closer to identified environmental sources (indicated with red triangles). Households near agricultural or industrial facilities tend to present more complex pesticide profiles. The heatmap overlay, based on Spearman’s rho values, indicates the strength and direction of correlations between soil pesticide concentrations and distance to these sources.

**Fig. 2:**
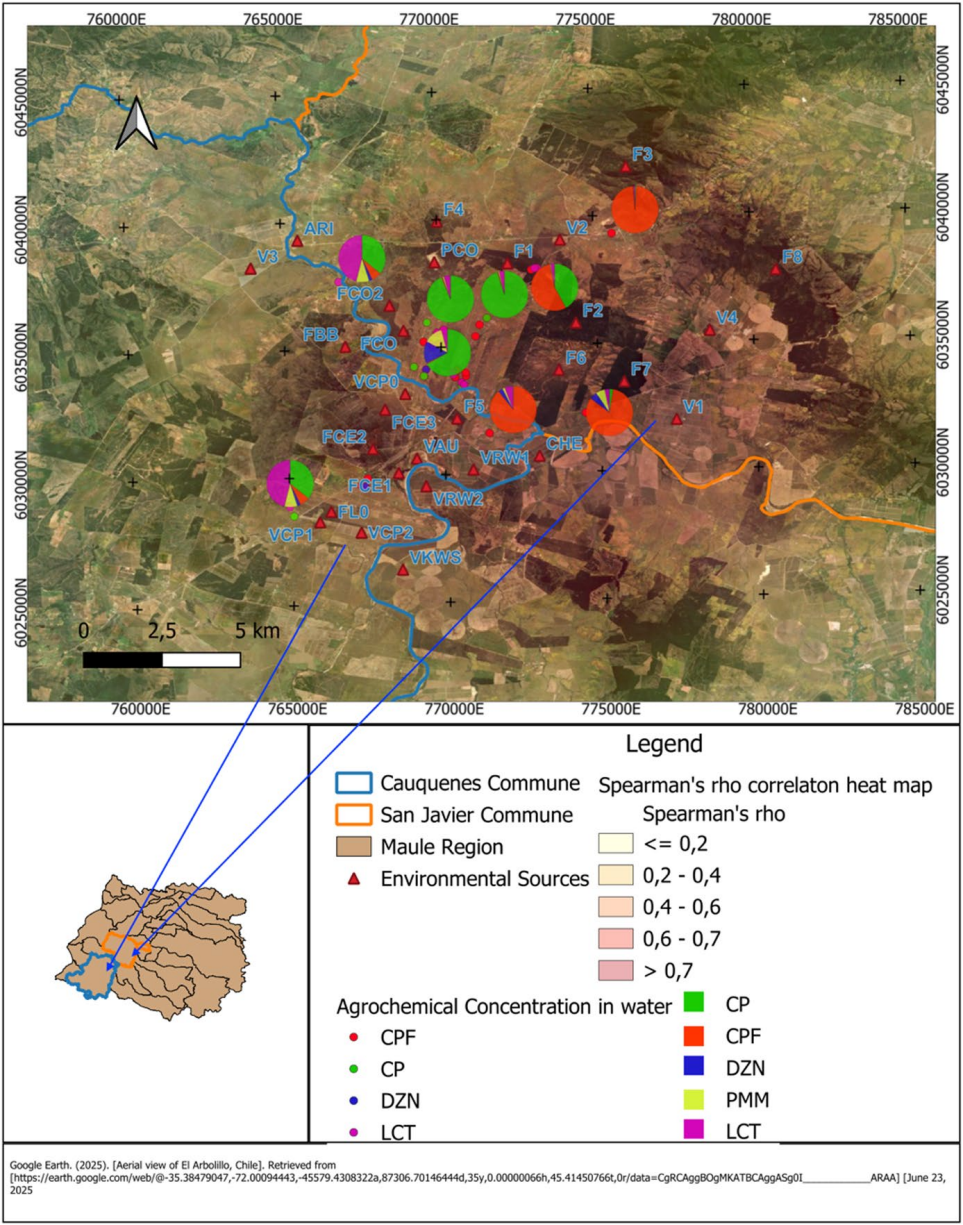
Spatial distribution of agrochemical residues in well water samples and correlation with proximity to environmental sources in rural Maule*. Map source: Google Earth satellite imagery (© Google; aerial imagery of El Arbolillo, Maule Region, Chile; accessed June 23, 2025). Map creation and editing: The authors created the maps using QGIS (version 3.34.4 ‘Prizren’; https://qgis.org). All spatial layers, symbols, and analyses were generated by the authors. *Note: The map shows the concentration of chlorpyrifos (CPF), diazinon (DZN), pirimiphos-methyl (PMM), cypermethrin (CP), and lambda-cyhalothrin (LCT) in well water samples collected from rural households in the communes of Cauquenes and San Javier. Each symbol represents a household with available well water data; smaller points correspond to dwellings with low-level detections and are shown in a distinct color to differentiate them from households with more frequent or higher concentrations. The pie charts illustrate the proportional contribution of each pesticide to the total residue load detected in each well. The heat map represents the magnitude of Spearman’s rho correlation between the distance from each household to nearby agricultural, forestry, or livestock facilities and the concentration of pesticides detected in well water. Darker tones indicate stronger correlations, meaning that pesticide levels tend to increase as households are located closer to these environmental sources. This visualization supports the interpretation that geographic proximity is an important determinant of environmental exposure in these rural communities.

Figure 2 presents the spatial distribution of pesticide residues detected in well water samples from rural households in the communes of Cauquenes and San Javier, Maule Region. Each pie chart corresponds to a sampling site and illustrates the relative contribution of detected agrochemicals CPF, CP, DZN, PMM, and LCT.

The heatmap represents Spearman’s rho values, indicating the strength of the correlation between pesticide concentrations and residential proximity to environmental sources (marked with red triangles). Darker areas indicate stronger positive correlations (ρ > 0.7), while lighter areas denote weak or null associations (ρ ≤ 0.2).

The spatial distribution reveals that households situated closer to environmental sources tend to exhibit higher concentrations of chlorpyrifos and cypermethrin in water samples. In contrast, households located farther from such sources generally show lower residue levels, consistent with distance-based attenuation of environmental inputs.

Table 4 presents the results of robust multiple linear regression models examining associations between mental health and quality-of-life outcomes and selected sociodemographic and environmental pesticide co-exposure variables.

**Table 4:** Robust multiple linear regression estimates for associations between environmental concentrations of organophosphate and pyrethroid pesticides, sociodemographic characteristics, and mental health and health-related quality-of-life outcomes (*n* = 82).

Variable	*R*²	β coefficient	*p*-value	95% CI Lower	95% CI Upper
*Depression Symptoms (CES-D)*	0.220				
- Sex		−3.528	0.091	−7.481	0.425
- Cypermethrin (water)		0.913	0.045	0.193	1.632
- Cypermethrin (soil)		−3.661	0.015	−6.990	−0.332
- Pirimiphos-methyl (soil)		21.291	0.032	1.784	40.797
- Chlorpyrifos (water)		0.180	0.044	0.016	0.345
- Pirimiphos-methyl (water)		−5.445	0.079	−12.359	1.470
- Chlorpyrifos (soil)		−0.587	0.084	−1.242	0.069
*Depression & Anxiety (GHQ-12*)	0.175				
- Sex		−1.164	0.044	−2.295	−0.032
- Cypermethrin (water)		0.324	0.002	0.124	0.525
*Physical Health Quality (SF-12 Physical)*	0.286				
- Sex		16.442	0.007	4.719	28.165
- Age		−0.608	0.004	−1.013	−0.203
- Education level		13.098	0.035	0.944	25.252
- Body Mass Index (BMI)		−1.254	0.053	−2.527	0.018
- Chlorpyrifos (water)		−0.467	0.015	−0.841	−0.092
*Mental Health Quality (SF-12 Mental)*	0.253				
- BMI		−0.935	0.186	−2.331	0.462
- Pirimiphos-methyl (soil)		21.142	0.036	1.396	40.888
- Lambda-cyhalothrin (soil)		−15.637	0.020	−28.707	−2.566
- Cypermethrin (water)		−2.113	0.074	−4.433	0.207
- Chlorpyrifos (water)		−0.713	0.016	−1.288	−0.137
- Pirimiphos-methyl (water)		27.399	0.201	−14.992	69.789
- Lambda-cyhalothrin (water)		−8.077	0.252	−22.040	5.885
*Overall Quality of Life (SF-12 Total)*	0.374				
- Age		−0.597	0.005	−1.009	−0.185
- Sex		14.962	0.014	3.136	26.787
- BMI		−1.252	0.056	−2.538	0.035
- Education level		12.349	0.051	−0.028	24.725
- Pirimiphos-methyl (soil)		15.402	0.099	−2.968	33.771
- Lambda-cyhalothrin (soil)		−15.129	0.017	−27.420	−2.837
- Chlorpyrifos (water)		−0.728	< 0.001	−1.112	−0.344
*Positive Affect (PANAS +)*	0.227				
- Age		0.137	0.007	0.039	0.234
- Per capita income		−1.36	0.012	−4.67	−2.25
- Chlorpyrifos (soil)		−0.114	0.036	−0.221	−0.008
- Time in the area		−0.073	0.039	−0.141	−0.004
*Negative Affect (PANAS -)*	0.192				
- Alcohol consumption		0.228	0.414	−0.325	0.781
- Diazinon (soil)		−1.025	0.144	−2.411	0.360
- Diazinon (water)		−1.036	0.214	−2.684	0.612
- Chlorpyrifos (soil)		−0.059	0.337	−0.181	0.063
- Time in the area		−0.046	0.207	−0.118	0.026

Depressive symptoms (CES-D) were significantly associated with pirimiphos-methyl concentrations in soil and with cypermethrin and chlorpyrifos concentrations in water, while an inverse association was observed for cypermethrin concentrations in soil. No statistically significant associations were observed for the remaining exposure variables. Psychological distress (GHQ-12) was higher among women and increased with higher cypermethrin concentrations in water. Physical health-related quality of life (SF-12 Physical*)* was higher among men and participants with higher educational attainment, and lower with increasing age and higher chlorpyrifos concentrations in water. Mental health-related quality of life (SF-12 Mental) was positively associated with pirimiphos-methyl in soil and inversely associated with lambda-cyhalothrin in soil and chlorpyrifos in water. Overall quality of life (SF-12 Total) decreased with age and with lambda-cyhalothrin in soil and chlorpyrifos in water, and was higher among men. Positive affect (PANAS +) increased with age and decreased with lower income, longer residence time, and higher soil chlorpyrifos concentrations. No statistically significant associations were observed for negative affect (PANAS -).

## Discussion

This study examined associations between environmental pesticide exposure and psychological symptoms among rural populations not engaged in agricultural labor in the Maule Region of Chile. In contrast to previous national research that has focused primarily on occupational exposure in agricultural settings^23,24^, the present findings pertain to residents living near agro-industrial, forestry, or livestock activities, where environmental exposure pathways may also be present.

Environmental analyses identified multiple pesticide compounds in soil and drinking water, with chlorpyrifos, cypermethrin, diazinon, and pirimiphos-methyl detected most frequently. Higher concentrations of chlorpyrifos and cypermethrin were significantly associated with increased levels of depressive symptoms, anxiety, and psychological distress, as well as with lower scores in mental health-related quality of life. These observations are consistent with previous studies conducted mainly in occupationally exposed populations, which have reported associations between exposure to organophosphates and pyrethroids and neurobehavioral or affective outcomes^2,10,26^.

The regression models explained between 17% and 37% of the variance in mental health and quality-of-life outcomes. These values are comparable to those reported in studies conducted in small rural populations, where psychological outcomes are shaped by multiple environmental, social, and individual factors. In this context, pesticide residues should be interpreted as one component within a broader set of determinants rather than as an isolated contributor to mental health variability^2^.

### Environmental concentrations and regulatory context

Pesticide concentrations measured in drinking water exceeded international guideline values for several compounds^27^. Chlorpyrifos was detected in 39.47% of samples, with maximum concentrations of 61.57 µg/L, above the guideline value proposed by the World Health Organization and the U.S. Environmental Protection Agency for drinking water (30 µg/L)^27^. Diazinon was detected in 22.37% of samples, with concentrations up to 4.58 µg/L, which is higher than commonly cited international reference values (0.1 µg/L)^27^. These concentrations are consistent with intensive agricultural use and the environmental persistence of these compounds reported in agricultural contexts, which may facilitate exposure through contaminated water and food sources^19,28^.

From a regulatory perspective, the Chilean drinking water standard NCh409/1 does not define compound-specific limits for chlorpyrifos or diazinon, although it mandates control of chemical substances that may pose health hazards^29^. In addition, no regulatory thresholds are established for groundwater used for household consumption or irrigation in areas without access to centralized water supply systems. The concurrent presence of cypermethrin (21.95%, up to 8.62 µg/L), pirimiphos-methyl (14.63%, up to 1.25 µg/L), and lambda-cyhalothrin (9.76%, up to 4.05 µg/L) indicates mixed contamination by organophosphate and pyrethroid compounds. For lambda-cyhalothrin, transport via surface runoff remains a plausible explanation for its occurrence in water despite strong soil adsorption^30,31^. Intensified pesticide application for fly control associated with pig farming activities may contribute to cumulative environmental loading in the area, particularly in rural communities relying on well water or irrigation sources.

### Matrix-specific distribution and physicochemical considerations

The distribution of pesticide residues across environmental matrices was consistent with known physicochemical properties of the detected compounds. Organophosphates such as chlorpyrifos and diazinon have moderate solubility and lower soil sorption than pyrethroids, which may contribute to their more frequent detection in well water. In contrast, cypermethrin and lambda-cyhalothrin are characterized by high hydrophobicity and strong adsorption to soil organic matter, and were more commonly detected in soil samples. Similar media-specific patterns have been described in environmental monitoring studies conducted in agricultural regions^32^.

### Associations with depressive symptoms and psychological outcomes

Depressive symptomatology varied according to pesticide class and environmental matrix. Positive relationships were observed for selected organophosphates, including pirimiphos-methyl in soil and chlorpyrifos in water, while patterns for cypermethrin differed by matrix, with positive estimates in water and inverse estimates in soil. Differences across matrices may be related to variation in residue distribution, detection frequency, or exposure contexts rather than to consistent directionality across compounds^33,34^. The concurrent detection of organophosphate and pyrethroid residues in environmental samples indicates the presence of mixed environmental exposures, which should be considered when interpreting these findings^35,36^.

Previous studies have reported associations between chronic or repeated exposure to organophosphates and depressive or neurobehavioral symptoms, particularly in agricultural populations^2,37,38^. Chlorpyrifos has been among the most frequently studied organophosphates regarding mental health indicators, largely due to its widespread use and persistence in environmental matrices. Associations reported in previous studies have been described in both occupational and residential contexts^16,18^.

### Residential proximity and non-occupational exposure pathways

Pesticide concentrations in soil and water were higher in households located closer to agricultural or industrial sources. This observation is consistent with established non-occupational exposure pathways, including pesticide drift, groundwater infiltration, and contact with contaminated surfaces. Previous research in the same region reported higher urinary organophosphate metabolite concentrations among populations residing near cultivated areas, supporting residential proximity as a determinant of environmental exposure^23,39^.

### Methodological considerations and analytical strengths

The use of Huber-IRLS regression reduced the influence of extreme values, which are common in environmental concentration data with skewed distributions^40^. Modeling psychological outcomes as continuous variables allowed the assessment of variation across the full distribution, avoiding information loss associated with dichotomization or arbitrary cutoffs^41^. In addition, the psychological instruments applied have been previously validated in Chile, supporting their psychometric appropriateness in this population.

### Policy relevance and regional implications

As regulatory frameworks evolve, measures to reduce residential exposure to pesticide residues may be considered in rural settings. These may include improvements in well sanitation, infrastructure to limit surface runoff, and the use of water filtration systems capable of reducing pesticide concentrations. In parallel, strengthening environmental health surveillance during periods of high pesticide application could support exposure monitoring in affected communities. Historical records from a 2019 judicial case indicate that the pig farming operation in the study area used pesticides such as diazinon, cypermethrin, and pirimiphos-methyl for vector control^25^, indicating long-standing environmental presence of these compounds in the area.

In the Latin American and Caribbean literature, evidence on pesticide exposure among communities near large-scale pig production operations is limited, particularly regarding recurrent increases in fly populations and associated vector control practices. Few studies have examined combined exposure scenarios arising from multiple agro-industrial activities that apply different classes of pesticides within the same territory. The present findings document environmental pesticide residues in an agroecological rural community where residents are not engaged in agricultural labor, yet are environmentally exposed due to the proximity of intensive agricultural and livestock activities^13,24–26,42^.

Chlorpyrifos was repeatedly detected in environmental samples. International restrictions on its use have expanded in response to evidence of neurotoxicity; however, this compound remains authorized in Chile^25^. Several international health agencies have recommended the progressive withdrawal of highly hazardous pesticides as a preventive measure. International experience with the implementation of regulatory measures suggests that population exposure to highly hazardous pesticides can be reduced without compromising agricultural production, providing a relevant policy context for interpreting these findings^43^.

### Environmental matrices and implications for future exposure assessment

The use of environmental matrices allows characterization of spatial patterns and compound-specific profiles of pesticide contamination at the community level. Although individual biomarker data were not collected in this study, previous research conducted in the same region has reported correspondence between environmental pesticide concentrations and urinary biomarkers. In this context, soil and water measurements can inform the design of future studies incorporating individual-level biomonitoring, including the prioritization of target compounds and the definition of exposure windows.

### Limitations

Several limitations should be considered when interpreting these findings. First, the cross-sectional design does not allow conclusions about directionality, as the temporal sequence between environmental exposure and the onset of mental health symptoms cannot be established; consequently, reverse causation cannot be excluded. Second, the absence of individual-level biomonitoring limits the assessment of internal dose and interindividual variability in absorption and metabolism. Third, the geographically localized scope of the study restricts the extent to which the results can be extrapolated to other rural contexts with different environmental and agricultural characteristics. In addition, dietary intake, a relevant pathway for chronic pesticide exposure in rural populations, was not evaluated. Although household insecticide use was collected, these exposures were not fully accounted for in the statistical models, potentially leading to incomplete control of confounding factors.

Contextual variables such as perceived environmental risk, social support networks, and access to health services were not evaluated, despite their potential to modify associations between environmental exposure and mental health outcomes. Within these constraints, the observed associations are consistent with previous epidemiological studies and align with regional surveillance data documenting acute pesticide poisonings in the study area.

Another methodological consideration relates to the treatment of values below the analytical limit of detection. For compounds with low detection frequencies, such as pirimiphos-methyl in soil and lambda-cyhalothrin in water, a proportion of observations were imputed using LOD/√2. This procedure reduces bias compared with assigning zero or excluding non-detects, but it increases uncertainty and decreases the effective variability of those exposure measures. Consequently, statistical power is reduced, and effect estimates for these compounds may be attenuated toward the null; findings involving pesticides with low detection frequencies should therefore be interpreted as exploratory.

### Future research directions

Future research would benefit from longitudinal designs to assess mental health trajectories in relation to cumulative environmental pesticide exposure. The incorporation of biomarkers, such as urinary dialkyl phosphates, together with improved geospatial exposure modeling, would strengthen exposure characterization. Further studies should also consider contextual factors, including psychosocial stress, risk perception, and access to healthcare, which may influence the relationship between environmental exposure and mental health outcomes.

## Recommendations

Enhancing environmental health monitoring in rural areas with limited connectivity would support the identification of pesticide residues in environmental matrices. Periodic community-based tracking of soil and drinking water can help detect contamination, particularly in households reliant on private wells. Community involvement in sampling and reporting processes can contribute to transparency and support local engagement in environmental decision-making.

Measures to protect domestic water sources may also reduce potential exposure. Actions such as routine well maintenance, the installation of protective structures, and the use of affordable water filtration systems could limit contamination while regulatory frameworks are updated. In parallel, municipal and regional authorities may reinforce oversight of agricultural and livestock operations to ensure compliance with existing environmental standards, including waste management, vector control practices, and pesticide application procedures^5^.

Promoting safer domestic pest control practices and targeted education on environmental health issues may help reduce cumulative exposure. Strengthening coordination between communities, primary healthcare services, and environmental agencies could improve communication and facilitate access to technical guidance and timely responses^36^.

Given the associations observed between environmental pesticide residues and mental health indicators, incorporating environmental exposure considerations into rural mental health programs may help identify households that could benefit from additional support. Coordinated efforts among public institutions, local communities, and agro-industrial stakeholders could support risk-reduction strategies in these rural contexts^2^.

## Methods

This observational cross-sectional study was conducted in rural areas of the San Javier and Cauquenes communes in the Maule Region of Chile. The selected areas are characterized by proximity to multiple sources of environmental pesticide exposure, including a large-scale pig farming facility, vineyards, croplands, and forestry operations. These sites have documented histories of intensive agrochemical use and have been associated with persistent socio-environmental conflicts^25^.

The target population comprised adult residents (≥ 18 years) living near the area known as “El Arbolillo,” where the San Agustín del Arbolillo pig production facility, authorized in 2008 for 10,000 sows, is a focal point of environmental controversy^25^. Households located within a 10-kilometer radius of the industrial complex were considered eligible. Participants were randomly selected from registries provided by local community organizations. Continuous residence in the study area for at least 12 months was required for inclusion.

Exclusion criteria were defined to reduce potential confounding. Individuals with a prior diagnosis of neurological disorders or with harmful alcohol consumption patterns, defined as a daily intake exceeding 40 g for women or 60 g for men^44^, were not eligible to participate.

Sample size estimation was informed by a prior study conducted in the Coquimbo Region that reported statistically significant differences in mental health outcomes between individuals environmentally exposed and not exposed to pesticides^13^. That study observed lower mean levels of psychological well-being among exposed participants, providing an empirical basis for estimating an expected difference between groups. Based on these parameters, a minimum sample of 60 participants (30 per exposure group) was required to detect differences in mental health outcomes with 80% statistical power and a two-sided significance level of 5%. To address potential non-response, missing data, or exclusions during fieldwork, an oversampling margin of 25% was applied. Accordingly, the target sample size was set at 80 participants.

This study was approved by the Scientific Ethics Committee of the Universidad Católica del Maule, Chile (Approval Acta N°112/2022; amendment Acta N°165/2023). All methods were carried out in accordance with the relevant guidelines and regulations, including the Declaration of Helsinki, the International Ethical Guidelines for Health-related Research Involving Humans (CIOMS), and Chilean Law 20.120, which regulates scientific research involving human beings. Written informed consent was obtained from all participants after a detailed explanation of the study and with a guarantee of complete confidentiality. All participants were adults, not members of vulnerable or captive populations, and participation was entirely voluntary. No coercion was applied, and participants provided their consent freely and with a complete understanding of the study.

Between December 2023 and January 2024, environmental samples of healthy water and peridomestic soil were first collected from participants’ households. Subsequently, standardized psychosocial instruments were individually administered to assess mental health outcomes. Data collection was carried out both at a designated community facility and in participants’ homes. The field team comprised trained professionals, including psychologists, physicians, biochemists, biologists, and other specialists.

### Pesticide exposure assessment

Environmental exposure was evaluated through the analysis of pesticide residues in drinking water and soil samples:

- *Water samples*: Drinking water was collected from household wells and rural supply points. All samples were preserved at 4 °C (± 2 °C) during transport and storage to ensure analyte stability. Extraction followed a QuEChERS-based protocol adapted for water matrices, involving acetonitrile partitioning and dispersive solid-phase extraction. Analytical detection of chlorpyrifos, diazinon, cypermethrin, and permethrin was performed using gas chromatography coupled with tandem mass spectrometry (GC-MS/MS). All water analyses were conducted at the Bioprocess Laboratory of the Universidad Católica del Maule^45^.
- *Soil samples*: Surface soil (0–10 cm) was collected from peridomestic areas near agricultural and industrial sites, including vineyards, forestry plantations, and pig farms. Samples were sieved, air-dried, and stored at 4 °C (± 2 °C) before analysis. A modified QuEChERS method adapted for soil matrices was used for extraction, employing acetonitrile with buffering salts, followed by clean-up with PSA and C18 sorbents to minimize matrix effects. Quantification of chlorpyrifos, diazinon, and lambda-cyhalothrin was performed using GC-MS/MS at the Bioprocess Laboratory of the Universidad Católica del Maule^46,47^.
- *Georeferencing*: Geographic coordinates of each participant’s residence and nearby potential exposure sources (e.g., pig farms, vineyards, forestry plantations) were recorded directly in the field. The shortest straight-linear distance (in meters) between each residence and the nearest exposure source was calculated using Google Earth. For spatial visualization and map generation, QGIS software was used to construct georeferenced layers and illustrate proximity patterns between households and identified sources of environmental contamination. These distance values were incorporated as continuous variables in the exposure assessment.

### Mental health outcome assessment

Mental health outcomes were assessed using a battery of validated self-report instruments designed to evaluate depressive symptoms, anxiety, emotional states, psychological distress, and perceived quality of life:

- *CES-D scale*: The Center for Epidemiologic Studies Depression Scale is a 20-item instrument that measures the frequency of depressive symptoms during the past week. Items are scored on a 4-point Likert scale (0 to 3), yielding a total score from 0 to 60. Higher scores indicate greater symptom severity. The scale was analyzed as a continuous variable. The Chilean version has demonstrated high internal consistency (Cronbach’s α > 0.85)^48^.
- *Positive and negative affect schedule (PANAS)*: Comprising two subscales of 10 items each, the PANAS assesses the intensity of positive (PA) and negative affect (NA). Items are scored on a 5-point Likert scale (1 to 5), with subscale scores ranging from 10 to 50. Higher scores show stronger affective states. The Chilean validation confirmed the two-factor structure with adequate internal consistency (Cronbach’s α = 0.91 for PA, 0.87 for NA)^49^.
- *General health questionnaire – 12 items (GHQ-12)*: The 12-item General Health Questionnaire (GHQ-12) is a self-administered scale that assesses psychological distress, including symptoms of anxiety, depression, and social dysfunction. It uses a 4-point Likert scale (0 to 3), with total scores ranging from 0 to 36, where higher scores indicate greater distress. In this study, GHQ-12 scores were analyzed as continuous variables to preserve statistical sensitivity. According to the Chilean validation (α = 0.902), the instrument showed high internal consistency and a two-factor structure, social dysfunction and psychological distress, that explained nearly 60% of the total variance^50^.
- *Short form health survey (SF-12 v2)*: This 12-item questionnaire assesses self-reported health across eight domains and generates standardized Physical (PCS) and Mental (MCS) component scores ranging from 0 to 100, with lower values indicating poorer health. For this study, the instrument was analyzed as a continuous variable. The Chilean version has shown good reliability (α = 0.899)^51^.

### Covariates

Additional instruments were applied to characterize participants’ sociodemographic, occupational, and behavioral profiles, and to adjust for potential confounders in the analysis:

- *General health and exposure history questionnaire*: A structured 26-item questionnaire, adapted from a previously validated instrument^52^, was used to collect information on sociodemographic characteristics (age, sex, education, marital status, per capita household income, and years of residence in the area), occupational history, disability status, and medical conditions potentially linked to pesticide exposure, including past episodes of acute poisoning. It also included items on occupational and domestic pesticide exposure. Anthropometric measurements were taken using a digital scale and stadiometer, with weight recorded in kilograms and height in centimeters.
- *Alcohol*,* smoking*,* and substance involvement screening test (ASSIST 3.0)*: This 8-item screening instrument assesses the level of risk associated with the use of ten psychoactive substances, including tobacco, alcohol, cannabis, cocaine, sedatives, and others. It evaluates both lifetime and recent use, adverse consequences, signs of dependence, and intravenous administration. Risk levels are categorized as low, moderate, or high. The Chilean validation demonstrated high internal consistency (Cronbach’s α = 0.91)^53^. In this study, the ASSIST was used as a covariate to control for behavioral factors that may confound associations between pesticide exposure and mental health outcomes.

All mental and physical health instruments were administered by trained health professionals with prior experience in community-based assessments. These professionals were specifically trained in the standardized administration of each questionnaire, ensuring consistent data collection and improving the reliability and comparability of responses across participants.

### Pesticide exposure assessment

Environmental pesticide exposure was characterized by measuring concentrations of selected organophosphate and pyrethroid compounds in peridomestic soil and drinking water collected at participants’ households. Residue analysis targeted chlorpyrifos, diazinon, pirimiphos-methyl, cypermethrin, and lambda-cyhalothrin, using gas chromatography coupled with tandem mass spectrometry (GC–MS/MS). Analytical quality was ensured through standard validation procedures, including assessment of recovery, precision, linearity, and detection limits, with correction for matrix effects when required. A detailed description of the extraction procedures and analytical validation is provided in the supplementary methods^45–47^.

### Analysis plan

Data were entered into a Microsoft Excel matrix using a double-entry procedure to minimize transcription errors. An exploratory analysis was conducted to identify missing data, outliers, duplicate entries, and to examine the distribution of all continuous variables.

Pesticide concentrations in soil (µg/kg) and water (µg/L) were processed using a substitution method for measurements below the analytical limit of detection (LOD). Non-detect values were replaced using the LOD divided by the square root of two (LOD/√2)^54^, an approach commonly used to handle measurements below the detection limit and to minimize bias in environmental quantification estimates (Table S1).

Given the heterogeneous detection frequencies across pesticides, the proportion of values below the LOD was examined before modeling. For compounds with very low detection rates (e.g., pirimiphos-methyl in soil, detected in only 7% of samples), the high fraction of imputed values increases uncertainty and reduces effective variability, limiting statistical power. Therefore, associations involving low-detection pesticides were interpreted cautiously and considered exploratory.

Categorical variables were described using absolute and relative frequencies. Continuous variables were summarized using means, medians, standard deviations, and interquartile ranges. Bivariate comparisons were performed with the Mann–Whitney U test, and correlations between continuous or ordinal variables were assessed using Spearman’s rank correlation coefficient.

To examine associations between pesticide concentrations, mental health outcomes, and residential proximity to exposure sources, six robust multiple linear regression models were fitted for each continuous mental health outcome: CES-D, GHQ-12, SF-12 Physical Component Score, SF-12 Mental Component Score, PANAS Positive Affect, and PANAS Negative Affect.

Covariates were selected based on prior evidence and theoretical relevance, including age, sex, education, marital status, per capita income, years of residence, occupational and domestic pesticide exposure, body weight, height, and substance-use risk.

Because exploratory analyses indicated the presence of outliers and heteroscedasticity, models were estimated using Huber’s M-estimator with iteratively reweighted least squares. Model comparison was conducted using adjusted R², inspection of residual distributions, and evaluation of multicollinearity. Only the models demonstrating the best goodness-of-fit and acceptable diagnostic performance were retained as final models.

Sensitivity analyses included excluding extreme values and comparing results with ordinary least squares estimates to assess the robustness of the findings. All analyses were conducted using Jamovi (v2.6) and Stata (v19.0).

## Supplementary Information

Below is the link to the electronic supplementary material.

Supplementary Material 1

## Data Availability

The datasets generated and/or analysed during the current study are not publicly available due to ethical and confidentiality restrictions. Data may be made available upon reasonable request from the corresponding author, María Teresa Muñoz-Quezada.
