# Supplementary material for: Environmental co-exposure to organophosphate and pyrethroid pesticides and mental health status in rural communities near an industrial pig farming facility
Source: Sci Rep. 2026 Feb 18;16:9769. doi: 10.1038/s41598-026-40098-1 (PMC13013602; doi:10.1038/s41598-026-40098-1)
Supplement: Supplementary file 1 — Supplementary Material 1 [file 41598_2026_40098_MOESM1_ESM.docx]

**Supplementary methods**

**QuEChERS extraction and analytical validation of pesticide residues in soil and water samples**

Environmental pesticide residues in peridomestic soil and well water samples were extracted using a QuEChERS-based methodology adapted for environmental matrices, following standardized protocols (EN 15662) with minor modifications to optimize recovery and matrix clean-up^45-47^.

***Soil samples***
Surface soil samples were air-dried, sieved to obtain a fine and homogeneous fraction, and stored at 4 °C until analysis. For extraction, 10 g of soil was weighed into 15 mL polypropylene centrifuge tubes. Subsequently, 20 mL of acetonitrile and 10 mL of ultrapure water were added, and samples were vortex-mixed for 1 minute. A QuEChERS extraction salt mixture (MgSO₄, NaCl, sodium citrate buffers; EN 15662) was then added, followed by vigorous shaking for 1 minute and sonication for 5 minutes at room temperature. Samples were centrifuged at 4000 rpm for 5 minutes.

An aliquot of 7 mL of the supernatant was transferred to a clean-up tube containing dispersive solid-phase extraction sorbents (MgSO₄, primary secondary amine [PSA], and C18) to reduce co-extracted matrix components. After vortex-mixing and centrifugation under the same conditions, 1 mL of the purified extract was evaporated to dryness at 50 °C. The residue was reconstituted in 1 mL of HPLC-grade acetonitrile, filtered through a 0.22 µm PTFE membrane, and transferred to autosampler vials for GC–MS/MS analysis.

***Water samples***
Well water samples were thawed at room temperature prior to extraction. A 10 mL aliquot of each sample was transferred to a 15 mL centrifuge tube, followed by the addition of 10 mL of HPLC-grade acetonitrile and QuEChERS extraction salts (EN 15662). The mixture was vortexed for 1 minute and centrifuged at 4000 rpm for 5 minutes to achieve phase separation.

A 7 mL sample of the acetonitrile layer was dispersive-cleanup using magnesium sulfate (MgSO₄) and primary-secondary amine (PSA) sorbents. Following vortex mixing and centrifugation, 1 mL of the cleaned extract was evaporated to dryness at 50 °C. The residue was then reconstituted in 1 mL of HPLC-grade acetonitrile, filtered through a 0.22 µm PTFE filter, and transferred to GC–MS/MS vials.

***Analytical validation***
The QuEChERS extraction procedure was analytically validated for both soil and water matrices. Mean recoveries ranged from 85.1% to 93.5% across all analytes, with intra-assay precision between 3.5% and 9.8% relative standard deviation (RSD). Calibration curves showed excellent linearity (R² ≥ 0.998) over working ranges of 0.5–250 µg/L for water and 1–500 µg/kg for soil. Matrix effects were observed for pyrethroids in soil; therefore, matrix-matched calibration was applied. Limits of detection (LOD) and limits of quantification (LOQ) were determined for each pesticide to characterize analytical sensitivity (Table S1). Overall, these results confirm the suitability of the QuEChERS–GC–MS/MS approach for quantifying organophosphate and pyrethroid residues in environmental soil and water samples^45-47^.

**Table S1.** Limits of detection (LOD), limits of quantification (LOQ), and substitution values (LOD/√2) for pesticide residues in soil (µg/kg) and water (µg/L) analyzed by GC–MS/MS

| **Pesticide** | **LOD** | **LOQ** | **LOD/√2** |
| --- | --- | --- | --- |
| Chlorpyrifos | 0.068 | 0.312 | 0.048 |
| Diazinon | 0.022 | 0.128 | 0.016 |
| Pirimiphos-methyl | 0.087 | 0.375 | 0.062 |
| Cypermethrin | 0.353 | 1.635 | 0.250 |
| Lambda-cyhalothrin | 0.454 | 2.515 | 0.321 |
